# Thiamine Deficiency Masquerading As Guillain-Barré Syndrome

**DOI:** 10.7759/cureus.78310

**Published:** 2025-01-31

**Authors:** Karima Benameur, Karen Clarke

**Affiliations:** 1 Department of Neurology, Emory University, Atlanta, USA; 2 Division of Hospital Medicine, Emory University, Atlanta, USA

**Keywords:** acute neuromuscular paralysis, acute neuropathy, nutritional deficiency, thiamine deficiency, vitamin b1 deficiency

## Abstract

Thiamine (vitamin B1) deficiency may be associated with serious neurological manifestations, including peripheral neuropathy, paralysis, confusion, paresthesia, pain, dysarthria, and nystagmus. The primary etiology of thiamine deficiency is consumption of a diet deficient in thiamine. In the United States, the most common cause of thiamine deficiency is alcohol use disorder due to the poor dietary intake that is often associated with this diagnosis. Thiamine deficiency also develops at an increased frequency in patients who have had gastric bypass surgery. Other medical conditions less commonly associated with thiamine deficiency include hyperemesis gravidarum, diabetes, and malnutrition outside of alcohol use disorder.

Prompt recognition of thiamine deficiency is important since it is readily treatable, and prompt treatment increases the likelihood of favorable clinical outcomes. On the other hand, delayed diagnosis and treatment of severe thiamine deficiency can lead to permanent neurological deficits or may even be fatal. Since clinicians often do not consider thiamine deficiency in patients without a history of alcohol use disorder, this diagnosis is often either delayed or missed altogether.

This case series presents four patients with acute neuropathy due to thiamine deficiency (normal range >70-180 nmol/L) and with corresponding neurophysiological and electromyographical data. It highlights the need to consider thiamine deficiency in all patients with acute to subacute lower extremity weakness or numbness.

## Introduction

Acute neuropathies presenting as quadriparesis progressing over days to weeks is a relatively common neurological presentation. The typical history of ascending weakness beginning in the legs and progressing to the arms, at times affecting the respiratory and bulbar muscles, is classically suggestive of Guillain-Barré syndrome. A detailed history, neurological examination, and diagnostic studies are useful in localizing the site of the lesion along the neuraxis (peripheral nerves, muscles, neuromuscular junction, spinal cord) and in establishing the correct diagnosis with important implications for treatment and prognosis. Guillain-Barré syndrome is a rare diagnosis; that is, there are 3000-6000 new cases per year in the United States and an estimated 100,000 new cases annually worldwide. It is the most common cause of acute neuropathy worldwide, and it is also responsible for most cases of respiratory muscle weakness associated with neuromuscular disorders [1,2].

Thiamine (vitamin B1) deficiency, classically called beriberi, can be associated with serious neurological (dry beriberi) or cardiac (wet beriberi) manifestations [3]. It is considered widely prevalent (up to 25% of mothers). However, it is underdiagnosed in low- and middle-income countries where the diet predominantly comprises polished white rice or milled grains in Southeast Asia and Africa [4]. Although less prevalent in developing countries, it remains widely underdiagnosed in the United States and Europe owing to its varied clinical presentations, with some reports of deficiency in up to 33% of patients with heart failure [3]. The most recognized neurological complication is Wernicke encephalopathy, classically presenting with the triad of confusion, ophthalmoplegia, and ataxia. Peripheral neurological complications, however, are less well recognized [3].

This case series presents four patients with acute neuropathy due to thiamine deficiency outside of the classically recognized scenario in patients with alcohol use disorder. This paper aims to highlight acute neuropathies due to thiamine deficiency to increase recognition in clinical practice.

## Case presentation

Case 1

A 24-year-old female, currently 33 weeks pregnant, was transferred from an outside hospital for evaluation of "Guillain-Barré syndrome not responding to usual treatments." Throughout her pregnancy, she had intractable vomiting with a resulting 120-pound weight loss by the time of transfer to our institution.

Symptoms consisted of rapidly progressive right lower extremity weakness, which progressed two weeks later to involve her left lower extremity, leading to her requiring a walker for ambulation. In addition to her bilateral lower extremity weakness, she also developed urinary retention, felt like she was “drunk,” and had blurry vision. She denied having any upper extremity involvement, diplopia, confusion, vertigo, or any other neurological symptoms.

Her neurological exam was remarkable for symmetrical distal weakness in both lower extremities. The Medical Research Council (MRC) scale for muscle strength was 1-3/5 with reduced light touch and pinprick sensation to the mid-thigh and no ataxia and generalized hyporeflexia (1+ throughout).

Guillain-Barré syndrome was suspected at the outside hospital, given the rapidly progressive ascending sensorimotor symptoms and hyporeflexia. Magnetic resonance imaging (MRI) of the whole spine and cerebrospinal fluid (CSF) analysis were normal. She was given intravenous immunoglobulin therapy (2 g/kg split over five daily doses). Then, she was referred to our tertiary hospital for further work-up due to lack of response to treatment.

An inpatient electromyography/nerve conduction study (EMG/NCS) showed electrophysiologic evidence of an active and ongoing severe sensorimotor axonal polyneuropathy. Her thiamine level was 32 nmol/L (normal range >70-180 nmol/L). She was treated with high-dose IV thiamine supplementation and physical therapy.

Case 2

A 40-year-old female with a history of bipolar disorder, schizophrenia, epilepsy, and gastric bypass at the age of 16 years old was transferred from an outside hospital for evaluation of worsening ascending weakness over the course of three weeks. Her initial presentation consisted of a five-day history of bilateral lower extremity weakness, which abruptly developed the day after she underwent an outpatient colonoscopy. She had several falls, which prompted her presentation to the outside hospital. Further history provided by the patient included severe diarrhea for several weeks and poor oral intake for several months due to anorexia, resulting in a 50-pound weight loss.

Physical examination revealed a lethargic patient who was not following commands. Cranial nerves were intact except for a disconjugate gaze. Motor strength was severely reduced (MRC 0/5 in both lower extremities and 2/5 in both upper extremities). Sensory examination showed decreased localization in all extremities, and reflexes were trace all over with mute plantar responses.

MRI of the brain revealed hyperintensity in the mamillary bodies consistent with Wernicke’s encephalopathy. MRIs of the entire spine and CSF analysis were normal. Thiamine level was 22 nmol/L (normal range >70-180 nmol/L). EMG/NCS study showed electrophysiological evidence of an active and ongoing severe sensorimotor axonal polyneuropathy.

Case 3

A 48-year-old female with a history of hypertension, obesity, and gastric sleeve surgery nine months ago presented to the emergency department with abdominal pain, vomiting, and a one-month history of numbness and burning paresthesia affecting both legs and her left hand. Physical examination revealed preserved strength in all muscle groups, decreased temperature up to the mid-thighs bilaterally with significant allodynia affecting both feet, an ataxic gait, and trace reflexes all over with mute plantar responses. MRI of the spine and CSF analysis were normal. Thiamine level was low at 32 nmol/L (normal range >70-180 nmol/L). EMG/NCS showed electrophysiological evidence of a subacute and ongoing sensorimotor axonal polyneuropathy.

Case 4

A 43-year-old female with a history of psoriatic arthritis presented as a code stroke to the emergency room. The patient reported that she was in her usual state of health until she suddenly had trouble climbing stairs due to bilateral lower extremity weakness. By the next day, her bilateral lower extremity weakness had worsened so that she could not walk. Neurological exam was remarkable for mild confusion, bilateral internuclear ophthalmoplegia with vertical nystagmus, significant weakness in the legs (MRC 2/5) and arms (MRC 4-/5) with significant loss of proprioception and vibratory sense throughout, and significant appendicular ataxia. Reflexes were absent throughout, and toes were down-going bilaterally.

**Figure 1:**
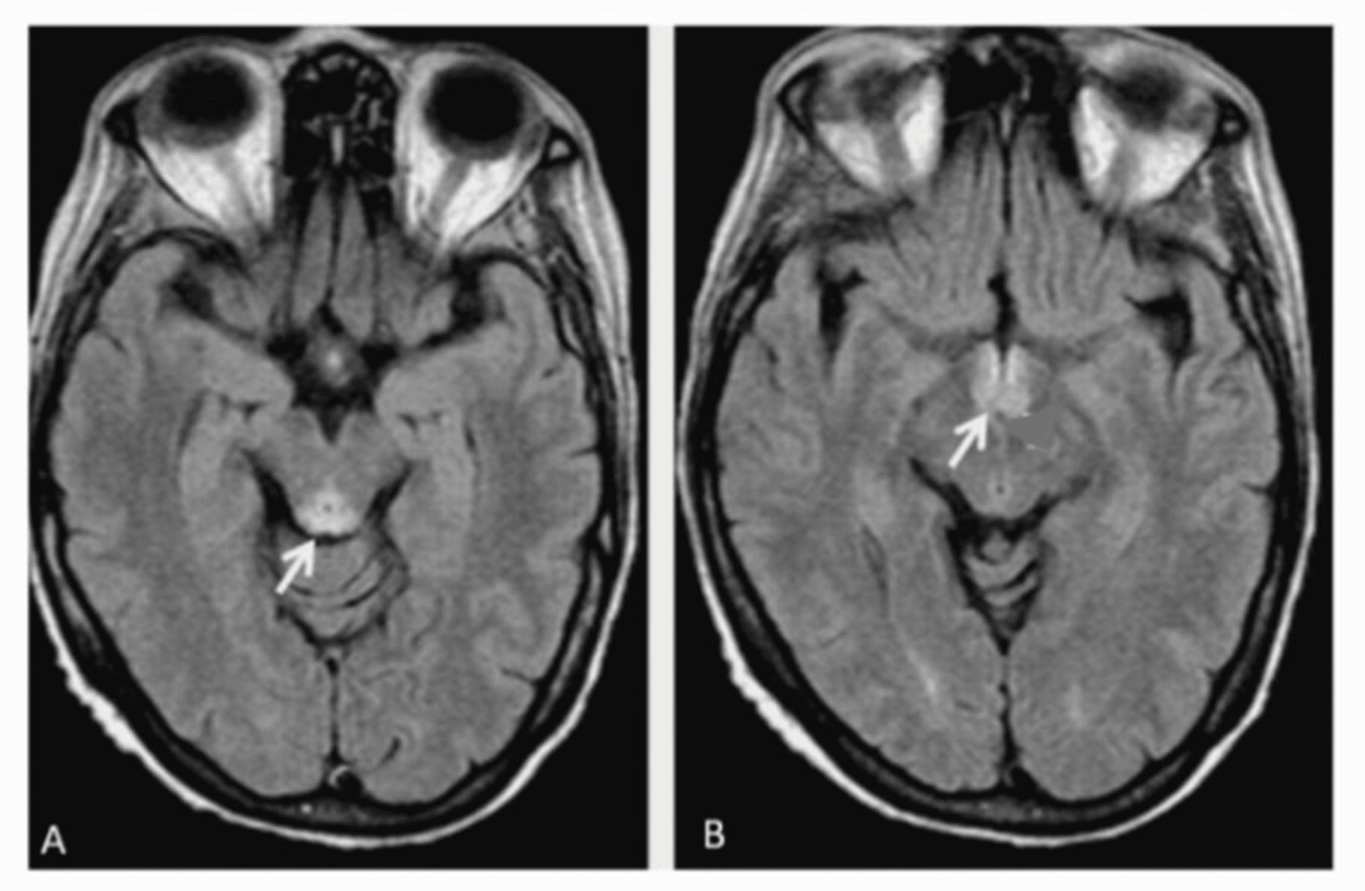
Brain MRI for Case 4 FLAIR images showing T2 hyperintensity in the periaqueductal gray (A) and mamillary bodies (B) as shown with arrows. MRI: magnetic resonance imaging, FLAIR: fluid-attenuated inversion recovery

The head computed tomography (CT) scan was normal. Brain MRI showed relatively symmetric abnormal T2/FLAIR hyperintensity in the bilateral medial thalami, mammillary bodies, and periaqueductal gray consistent with Wernicke encephalopathy (Figure 1). MRI of the spine and CSF analysis were normal. Her thiamine level was 38 nmol/L (normal range >70-180 nmol/L) (Table 1). She denied having a history of alcohol use disorder, and a negative urine ethyl glucuronide screen confirmed this. Systemic malabsorption workup revealed elevated levels of transglutaminase IgA antibodies (572 mg/dL, normal 68-408 mg/dL). EMG/NCS showed electrophysiological evidence of a subacute ongoing sensorimotor axonal polyneuropathy.

**Table 1:** Patient presentation, thiamine level, and EMG findings EMG: electromyography

	Presentation	Thiamine level (nmol/L) (normal >70–180 nmol/L)	EMG findings
Case 1	24-year-old pregnant female with hyperemesis gravidarum who presented with bilateral lower extremity weakness	32	Electrophysiological evidence for an active and ongoing, severe, sensorimotor, axonal polyneuropathy
Case 2	40-year-old female with a history of gastric bypass surgery who presented with ascending flaccid paralysis	22	Electrophysiological evidence for an active and ongoing, severe, sensorimotor, axonal, polyneuropathy
Case 3	48-year-old female with a history of celiac disease diagnosed as a result of this presentation presented with ascending weakness and ataxia	32	Electrophysiological evidence for an acute to subacute, active, and ongoing sensorimotor axonal polyneuropathy
Case 4	43-year-old female with high-calorie malnutrition who presented with bilateral lower extremity weakness	38	Electrophysiological evidence for an acute to subacute, active, and ongoing sensorimotor axonal polyneuropathy

## Discussion

Acute neuropathies are relatively rare and are often associated with autoimmune causes, such as Guillain-Barré syndrome. This case series emphasizes the need to broaden the differential diagnosis beyond autoimmune conditions and to consider causes of thiamine deficiency beyond alcohol use disorder. Clinical and electrophysiological evaluations are essential for localizing the lesion to the anterior horn cell, nerve roots, nerves, muscles, or neuromuscular junctions. While Guillain-Barré syndrome and ICU-related critical illness neuromyopathy are commonly recognized causes of acute weakness from neuropathy, other less recognized causes, such as nutritional deficiencies, can also cause acute neuropathies. In this case series, we present patients who developed acute and subacute weakness due to thiamine deficiency, presenting as acute neuropathy. We propose a practical approach to help avoid diagnostic delays and improve patient outcomes.

Thiamine is a vital water-soluble vitamin that is a key cofactor in maintaining normal nervous system functions. It is integral to energy production within mitochondria, particularly in the pathways of glycolysis, the citric acid cycle, and the pentose phosphate pathway. Thiamine also contributes to neurotransmitter synthesis and myelin production and has notable antioxidant effects. Thiamine deficiency can lead to neuronal damage and demyelination due to impaired energy production. Since the body does not produce thiamine naturally, it must be obtained from food sources such as poultry, beef, cereals, nuts, and beans. The recommended daily intake of thiamine is about 1.2 mg for men and 1.1 mg for women, slightly increasing to 1.4 mg during pregnancy or breastfeeding [3,4]. The body’s ability to store thiamine is limited, with excess amounts excreted after reaching approximately 30 mg, and the vitamin’s half-life is estimated at nine to 18 days. Consequently, deficiencies can develop rapidly, especially in countries where enriching foods is not a regular practice [3,4].

Clinical investigations typically involve the measurement of thiamine levels. Thiamine can be measured in various biological samples, including whole blood, erythrocytes, serum, plasma, and urine. Whole blood provides measurements of all three thiamine derivatives, with thiamine pyrophosphate (TPP) accounting for approximately 90% of circulating thiamine, 80% of which is found in erythrocytes. Free thiamine, thiamine monophosphate, and TPP are primarily detected in serum, plasma, and urine [4]. The most common method for measuring TPP in whole blood is liquid chromatography-tandem mass spectrometry (LC-MS/MS). In the United States, the reference ranges for LC-MS/MS TPP at Quest Diagnostics and LabCorp are 78-185 nmol/L and 66.5-200.0 nmol/L, respectively [5,6].

While whole-blood TPP measurements are convenient, they are subject to interference from variables such as hematocrit and hemoglobin levels. The most accurate assessments of thiamine status are obtained through direct measurement of erythrocyte TPP using high-performance liquid chromatography (HPLC) or indirectly through the erythrocyte transketolase activation test. In healthy individuals, reference ranges for TPP measured by HPLC are 70-180 nmol/L, with total thiamine levels ranging from 75-195 nmol/L [7].

Despite the widespread availability of dietary thiamine in affluent nations, thiamine deficiency remains a significant yet often overlooked concern. In developed countries, extensive industrial food processing frequently reduces thiamine content and other essential vitamins and nutrients. In 1940, the Committee on Food and Nutrition recommended fortifying flour with thiamine, niacin, riboflavin, and iron to promote nutrient sufficiency [8]. Since implementing this policy, consuming enriched and fortified products has become the primary means of achieving adequate nutrient intake in the United States. Consequently, there is a prevailing perception that nutrient deficiencies, including thiamine deficiency, are uncommon outside of cases of severe malnutrition or alcohol use disorder. This perception has led to the inconsistent assessment of thiamine levels in both clinical practice and nutritional surveys, which are used to inform public health policies.

The increasing consumption of processed foods, particularly simple carbohydrates that lack sufficient thiamine, is called "high-calorie malnutrition." Thiamine plays a crucial role in glucose metabolism, and an increase in carbohydrate consumption proportionally raises the dietary requirement for thiamine, with a minimum of 0.33 mg required per 1,000 kcal [9]. Consequently, despite high caloric intake, the nutritional quality of these diets is poor, often failing to meet the recommended dietary guidelines for micronutrient intake and thereby placing individuals at risk for micronutrient malnutrition [10]. For example, studies have shown that at least 29% of obese individuals undergoing bariatric surgery are thiamine deficient [11]. Although national registries of thiamine levels in the general population do not exist, it is safe to deduce that thiamine deficiency is more common than assumed, given the high levels of obesity in the country [12]. Therefore, rather than focusing solely on the recommended daily allowance of thiamine, it is essential to align its intake with both carbohydrate consumption and total caloric intake.

The most classically recognized cause of thiamine deficiency in the United States is alcohol use disorder, owing to the poor dietary intake and inadequate absorption that are associated with alcoholism [13]. Other less well-recognized causes include obesity surgery (even years after the surgery), hyperemesis gravidarum, end-stage renal disease (especially among those requiring renal replacement therapy), advanced HIV/AIDS, and loop diuretic use [4,13].

The classic clinical triad of Wernicke’s encephalopathy consists of mental status changes, ocular abnormalities (including nystagmus and ophthalmoplegia), and gait ataxia [3], with classic changes in the mamillary bodies and periaqueductal gray on imaging as shown in Figure 1. However, the complete triad may be present in as few as 10% of cases, and MRI brain findings while highly specific (93%) remain poorly sensitive (53%), making reliance on imaging, particularly in cases with peripheral nervous system-only presentation, unreliable [14-16]. A post-mortem investigation of 131 cases of Wernicke encephalopathy found that 80% of cases were missed, likely because only 16% presented with the classic triad, 44% had one or two of the three symptoms, and 19% had none [17]. A less recognized neurological sign of thiamine deficiency is neuropathy, which is discussed in this case series.

Thiamine deficiency also affects non-neurological systems such as seen in cardiac beriberi, a high output cardiac failure also called wet beriberi, and gastrointestinal beriberi [18]. Thus, relying on a clinical suspicion based on the sole criterion of the classical triad is inadequate and may lead to underdiagnosis.

In this case series, thiamine deficiency was caused by less commonly considered etiologies, that is, hyperemesis gravidarum, bariatric surgery, and high-calorie malnutrition in an obese patient. All cases presented underwent an extensive work-up, including brain and spinal cord imaging, EMG/NCS, lumbar puncture, and multiple blood tests before the diagnosis of thiamine deficiency was established. EMG/NCS studies typically show an axonal sensorimotor polyneuropathy pattern, worse in the lower extremities. CSF can help, at times, differentiate from autoimmune etiologies, such as in Guillain-Barré syndrome, if a cytoalbuminologic dissociation is absent. However, cytoalbuminologic dissociation is not a prerequisite for diagnosing Guillain-Barré syndrome. Prompt treatment of thiamine deficiency is associated with better clinical outcomes than later initiation of thiamine repletion. A delay in diagnosing thiamine deficiency can have a substantial long-term impact on a patient’s clinical course. Therefore, initiating treatment upon clinical suspicion and before laboratory results are available is important, given that it often takes several days before they are available [19]. There is no consensus on the thiamine supplementation route or dose of treatment due to the absence of robust clinical trials that evaluate both the optimal route of administration and dosage. Parenteral administration is generally recommended initially, as it achieves high cerebral concentrations due to passive diffusion across the blood-brain barrier. While clinical practice emphasizes the use of high-dose infusions for alcohol-dependent patients, dosage practices and duration of treatment outside this group tend to vary significantly, underscoring the need for more data to guide future treatment [20].

Limitations of this case series include the lack of availability of treatment outcomes since it typically takes substantial time to observe positive results from thiamine repletion, and this case series was from an inpatient cohort reported by hospitalists. Another limitation of this case series is the relatively small number of patients included; however, the subject matter is generalizable to large patient populations.

## Conclusions

Thiamine deficiency can cause serious neurological complications, including acute neuromuscular paralysis. Early recognition and treatment of thiamine deficiency are associated with improved clinical outcomes compared to a delayed diagnosis. Therefore, it is imperative for clinicians to promptly consider thiamine deficiency in all patients who present with acute neuropathy, including those who do not have a history of alcohol use disorder. Routine testing of thiamine levels in high-risk populations should be considered to allow for the prompt diagnosis and treatment of thiamine deficiency.
